# Peripartum Investigation of Red Blood Cell Properties in Women Diagnosed with Early-Onset Preeclampsia

**DOI:** 10.3390/cells10102714

**Published:** 2021-10-10

**Authors:** Beata Csiszar, Gergely Galos, Simone Funke, Dora Kinga Kevey, Matyas Meggyes, Laszlo Szereday, Peter Kenyeres, Kalman Toth, Barbara Sandor

**Affiliations:** 1 Department of Anaesthesiology and Intensive Therapy, Medical School, University of Pécs, H-7624 Pécs, Hungary; 2 Szentágothai Research Centre, University of Pécs, H-7624 Pécs, Hungary; galosgergely@gmail.com (G.G.); meggyes.matyas@pte.hu (M.M.); szereday.laszlo@pte.hu (L.S.); kenyeres.peter@pte.hu (P.K.); toth.kalman@pte.hu (K.T.); sandor.barbara@pte.hu (B.S.); 3 1st Department of Medicine, Medical School, University of Pécs, H-7624 Pécs, Hungary; 4 Department of Obstetrics and Gynaecology, Medical School, University of Pécs, H-7624 Pécs, Hungary; funke.simone@pte.hu (S.F.); keveydorka@gmail.com (D.K.K.); 5 Department of Medical Microbiology and Immunology, Medical School, University of Pécs, H-7624 Pécs, Hungary

**Keywords:** early-onset preeclampsia, hemorheology, red blood cell aggregation, red blood cell deformability, erythrocyte

## Abstract

We investigated peripartum maternal red blood cell (RBC) properties in early-onset preeclampsia (PE). Repeated blood samples were taken prospectively for hemorheological measurements at PE diagnosis (n = 13) or during 26–34 weeks of gestation in healthy pregnancies (n = 24), then at delivery, and 72 h postpartum. RBC aggregation was characterized by M index (infrared light transmission between the aggregated RBCs in stasis) and aggregation index (AI—laser backscattering from the RBC aggregates). We observed significantly elevated RBC aggregation (M index = 9.8 vs. 8.5; AI = 72.9% vs. 67.5%; *p* < 0.001) and reduced RBC deformability in PE (*p* < 0.05). A positive linear relationship was observed between AI and gestational age at birth in PE by regression analysis (R^2^ = 0.554; *p* = 0.006). ROC analysis of AI showed an AUC of 0.84 (0.68–0.99) (*p* = 0.001) for PE and indicated a cutoff of 69.4% (sensitivity = 83.3%; specificity = 62.5%), while M values showed an AUC of 0.75 (0.58–0.92) (*p* = 0.019) and indicated a cutoff of 8.39 (sensitivity = 90.9% and specificity = 50%). The predicted probabilities from the combination of AI and M variables showed increased AUC = 0.90 (0.79–1.00) (*p* < 0.001). Our results established impaired microcirculation in early-onset PE manifesting as deteriorated maternal RBC properties. The longer the pathologic pregnancy persists, the more pronounced the maternal erythrocyte aggregation. AI and M index could help in the prognostication of early-onset PE, but further investigations are warranted to confirm the prognostic role before the onset of symptoms.

## 1. Introduction

Hypertensive disorders affect 5–10% of pregnancies worldwide and are still one of the leading causes of maternal and perinatal morbidity and mortality [1,2]. Preeclampsia (PE) is defined as new-onset hypertension accompanied by proteinuria and/or maternal organ and/or uteroplacental dysfunction manifesting at or after 20 weeks of gestation [3]. Early-onset PE develops before 34 weeks of gestation and has higher risks of maternal morbidity, perinatal death, and severe neonatal morbidity [4]. Short-term maternal consequences could be life-threatening, and women with a history of PE are more prone to suffering from cardiovascular diseases in later life [5,6]. The consequences also seriously affect the fetus since the placenta is not able to ensure adequate perfusion.

The pathophysiological explanation of early-onset PE is based on abnormal placentation, but the exact processes are still equivocal. It is suggested that impaired cytotrophoblast invasion into spiral arteries in the first trimester results in abnormal vascular remodeling with resistant vessels and elevated pressure [7]. The subsequent placental hypoxia leads to the release of pro-inflammatory factors, which might contribute to a pathological systemic endothelial response, characterized by increased capillary permeability, microvascular thrombosis, and sustained vascular hypertension, and consequently a manifestation of clinical signs and symptoms [8]. 

Red blood cells (RBCs) are the most abundant cells in the bloodstream affected by the deteriorated microcirculatory environment in PE, which leads to remarkable alterations in the erythrocyte morphology and the cell membrane [9]. The science of hemorheology investigates blood flow conditions, the physical properties of blood elements, including cellular and plasmatic components (i.e., RBC aggregation and deformability). Erythrocyte deformability is described as the ability of RBCs to adapt by deformation in response to mechanical forces to be able to cross over narrow capillaries. RBC aggregation means the rouleaux formation of RBCs under low flow conditions. Elevated RBC aggregation and decreased deformability can adversely affect tissue perfusion. The complex mechanisms of erythrocyte aggregation and deformability are discussed in more detail in the Supplementary Material (Supplementary Material for 1. Introduction section).

Altered RBC aggregation and deformability can be considered as the cause or the result of this complex circulatory disorder. Endothelial damage leads to microthrombus formation and erythrocyte fragmentation or hemolysis due to vascular mechanical forces in PE [9]. The blood viscosity at low shear conditions such as the placental intervillous space is mainly determined by RBC aggregation, and increased values potentially deteriorate placental microcirculation [10]. Enhanced RBC aggregation may have an additional increasing impact on peripheral vascular resistance, and the endothelial dysfunction may further increase blood pressure, thereby provoking a vicious cycle [11].

According to previous studies, maternal erythrocyte aggregation and deformability might deteriorate in PE leading to impaired blood flow at the uteroplacental cross-over [12]. However, limited information is available about the early-onset form.

## 2. Aim

Our research group intended to evaluate the peripartum maternal erythrocyte aggregation and membrane deformability alterations by repeated sampling in early-onset PE and to compare these with physiological changes in healthy pregnancies. 

A more precise understanding of the peripartum pathologic alterations of maternal RBCs would help to identify new therapeutic perspectives targeting the restoration of the erythrocyte function and improvement of oxygen-carrying capacity. In addition, we sought to identify aggregation and deformability parameters possessing the strongest ability to diagnose early-onset PE. The determination of these factors may help to identify which parameter changes should be given special attention even before the onset of symptoms.

## 3. Materials and Methods

### 3.1. Population

Thirteen non-smoking women diagnosed with early-onset PE based on the International Society for the Study of Hypertension in Pregnancy criteria and admitted to the Department of Obstetrics and Gynecology, University of Pécs, were involved in this prospective, case-control study [3]. 

**Figure 1:**
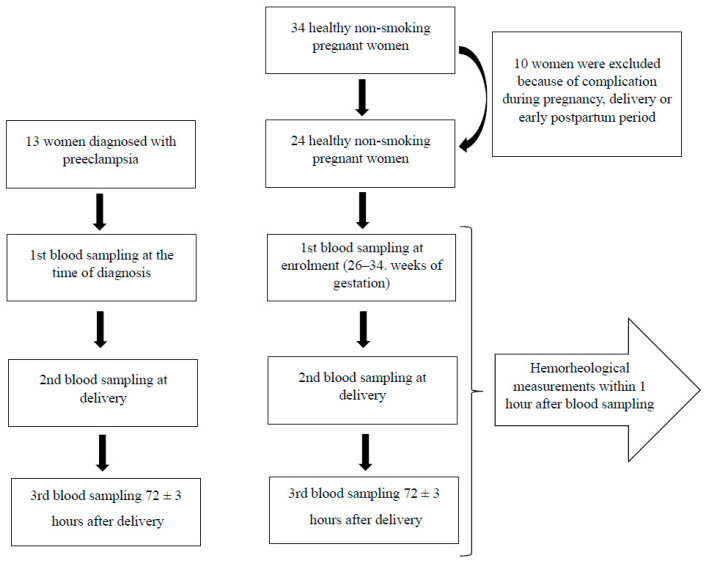
Process of recruitment and sample collection of women diagnosed with PE and healthy pregnant women.

The control group consisted of 24 healthy, non-smoking, age- and gestation-matched women. Before the enrolment, a physician excluded any relevant comorbidities (chronic hypertension, diabetes mellitus, cardiovascular, hematologic, gynecologic disorders). In the first phase, we recruited 34 women who were evaluated to be healthy and expected to have a pregnancy without complications. Finally, 10 women were excluded from the control group due to peripartum complications (Figure 1).

In both groups, exclusion criteria were twin pregnancy, intrauterine developmental abnormality of the fetus, intrauterine infection, severe maternal anemia, participation in another study, or lack of signed informed consent.

All women gave their written informed consent. The study protocol was approved by the Regional and Local Research Ethics Committee at the Medical School, University of Pécs (Reference number: 6942-PTE 2018). The study protocol conforms to the ethical guidelines of the 1975 Declaration of Helsinki.

### 3.2. Sample and Data Collection

Data collection included maternal anamnestic information, comorbidities, symptoms, gestational age at birth. We recorded laboratory parameters at diagnosis and within 72 h after delivery and maternal physical parameters (height, weight, body mass index-BMI, heart rate, blood pressure) at enrolment. Neonatal physical parameters (birth weight, length, head circumference, shoulder width) and the Appearance, Pulse, Grimace, Activity, and Respiration (Apgar) score determined immediately after the birth (Apgar 1) and 5 min later (Apgar 5) were also recorded.

Among patients, the first blood sample was drawn at diagnosis. Among controls, the first sample was collected at enrolment (26–34 weeks of gestation), later discussed as “initial” values. In both groups, blood samples were drawn within the 1st hour after delivery and 72 ± 3 h later. Every time, 2 × 6 mL of peripheral blood from antecubital veins were collected into EDTA-Vacutainer tubes. The hemorheological measurements were performed within one hour after sampling in the Hemorheological Laboratory of the University of Pécs under standardized conditions. Figure 1 summarizes the process of recruitment and sample collection. 

### 3.3. RBC Aggregation

RBC aggregation was determined with two different methods. The Myrenne aggregometer (model MA-1, Myrenne GmbH, Roetgen, Germany) measures infrared light transmission through the plasma gaps between the RBC aggregates between a transparent plate and a cone. The system rotates the injected 30 μL of blood for 10 s at high shear stress and disperses all pre-existing cell aggregates, then it instantly stops (M mode in stasis) or continues with reduced shear stress (M1 mode in low shear), which stimulates the aggregation and increases the light transmission. The aggregation is determined by the quantity of light transmission. The two dimensionless indices (M, M1) increase with enhanced erythrocyte aggregation [13]. More details about the measurement and the instrument can be found in the Supplementary Material (Supplementary Material for 3. Materials and Methods section; Supplementary Figure S1).

The Laser-assisted Optical Rotational Cell Analyzer (LORCA, R&R Mechatronics, Hoorn, Netherlands) determines the erythrocyte aggregation by detecting laser backscattering from the RBC aggregates. Briefly, a 1 mL blood sample is injected in the gap between the outer, rotating cylinder, and the inner, static cylinder of the instrument, and RBCs are disaggregated at a high shear rate until the motor rapidly stops. The intensity of reflected light is measured and plotted as a function of time. The aggregation index (AI) is calculated during the first 10 s of the measurement [14]. 

### 3.4. RBC Deformability

The erythrocyte deformability at different shear stresses was determined by LORCA as well. Briefly, a 20 μL blood sample is diluted in a viscous medium (polyvinylpyrrolidone) and injected between the cylinders of the instrument. A laser-diode is projected through the fluid, the light diffracts on the RBCs resulting in a diffraction pattern on a diaphragm, which will be analyzed. Increasing shear stresses elongate the RBCs, and the diffraction pattern changes from a circular to an elliptical shape. We could express RBC deformability as elongation index (EI) given at each shear stress [15]. More details about the aggregation and deformability measurements by LORCA can be found in the Supplementary Material (Supplementary Material for 3. Materials and Methods Section; Supplementary Figure S2).

### 3.5. Statistical Analysis

Statistical analysis was evaluated by IBM SPSS Statistics^®^ 27.0. Continuous variables are reported as mean ± standard deviation or medians and interquartile ranges, while categorical variables are presented as frequencies and percentages. After testing the normality by the Kolmogorov–Smirnov test, Mann–Whitney U-test or Student T-test and chi-square test were used to compare data between groups. Bivariate correlation analysis was performed calculating Spearman’s correlation coefficient (rho). Multiple regression analysis with various models including aggregation and deformability variables and maternal factors considering the principle of multicollinearity was performed to reveal which factors could predict gestational age at delivery. The diagnostic power of variables was assessed using the area under the curve (AUC) of the receiver operating characteristic (ROC) curve. The predicted probabilities were calculated from the combination of initial AI and M variables produced by binary logistic regression analysis. *p* ≤ 0.05 was considered statistically significant.

## 4. Results

### 4.1. Population Characteristics

Table 1 presents basic demographic and clinical data of the groups. The mean values of the maximum measured systolic and diastolic blood pressure in the PE group were 180 ± 18 mmHg and 112 ± 13 mmHg. Women with PE had significantly higher rate of preterm deliveries and infants with low birth weight, length, head circumference, shoulder width, intrauterine growth restriction (IUGR) and lower values of Apgar 1 and 5 as expected. In the PE group, all patients received antihypertensive medications (e.g., methyldopa), antenatal corticosteroid prophylaxis, and magnesium sulfate. The women in the control group did not take any medications regularly.

### 4.2. Routinely Measured RBC Laboratory Parameters

We found significantly elevated RBC count (4.29 ± 0.12 vs. 3.91 ± 0.05 T/L; *p* = 0.009), hematocrit (36.3% ± 2.7% vs. 34.3% ± 1.9%; *p* = 0.019), and hemoglobin (12.658 ± 0.260 vs. 11.791 ± 0.138 g/dL; *p* = 0.003), and significantly lower mean corpuscular volume (MCV) (84.71 ± 2.85 vs. 87.86 ± 4.56 fL; *p* = 0.037) in PE diagnosis compared to controls.

### 4.3. RBC Aggregation

**Figure 2:**
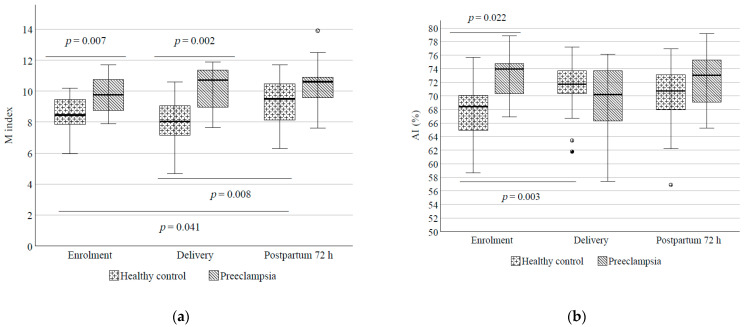
(**a**,**b**) M index and AI values reflecting RBC aggregation in PE and control group at the three investigated time points. Box plot diagrams show the median with interquartile ranges and the minimum and maximum values.

RBC aggregation measured with two different methods was significantly elevated in PE compared to controls. M indices were higher in PE at diagnosis (9.8 ± 0.4 vs. 8.5 ± 0.2; *p* = 0.007) and at delivery (10.7 ± 0.8 vs. 8.0 ± 0.4; *p* = 0.002) compared to those in healthy pregnancies. In controls, the M value increased until 72 h after birth compared to values at enrolment or delivery. This alteration was not observed in PE, where M values were continuously high (Figure 2a). We did not find a significant difference concerning M1 values.

The AI values were significantly higher at the initial measurement in PE than among controls (72.9% ± 3.5% vs. 67.5% ± 3.9%; *p* < 0.001) (Figure 2b). Investigating the values in control women showed that RBC aggregation significantly increased from the first blood sampling at enrolment to the time of delivery (AI: 67.5% ± 0.8% vs. 71.1% ± 1.0% *p* = 0.003), while this elevation was not visible in PE. 

To exclude the potentially disturbing impact of the mode of delivery, additional statistical analyses were performed involving all preeclamptic patients (n = 13; 100%) and nine women (37.5%) in the control group who had cesarean section. In addition, aggregation parameters were evaluated within the control group according to the mode of delivery. Our subgroup analyses showed no significant effect of the mode of delivery on the RBC aggregation results in the postpartum period (Supplementary Material for 4. Results Section; Supplementary Figures S3–S5).

**Figure 3:**
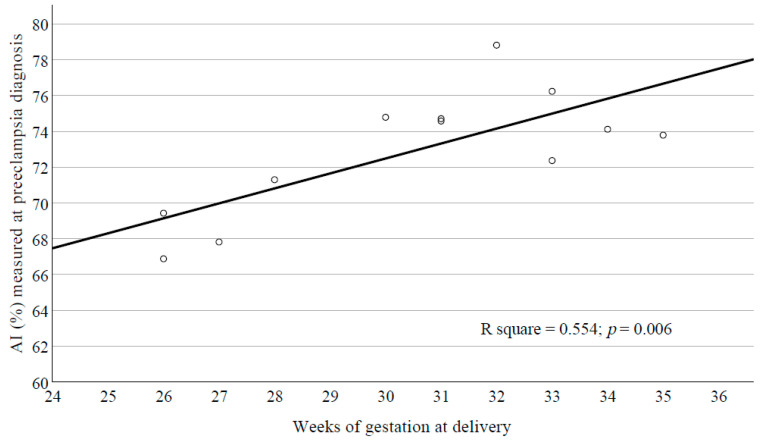
Linear regression analysis of initial AI measured at diagnosis of PE and weeks of gestation at birth.

A positive significant correlation was observed between initial AI measured at diagnosis and the weeks of gestation at delivery in PE (R^2^ = 0.554; *p* = 0.006) (Figure 3). A multiple regression analysis was run to predict gestational age at delivery from AI and EI at 5.33 shear stress and maternal systolic blood pressure at admission. These variables were statistically significant predicting gestational age at delivery, *p* < 0.029, R^2^ = 0.657. Out of the three variables, only AI added statistical significance to the prediction of gestational age at delivery, *p* < 0.010.

### 4.4. RBC Deformability

**Figure 4:**
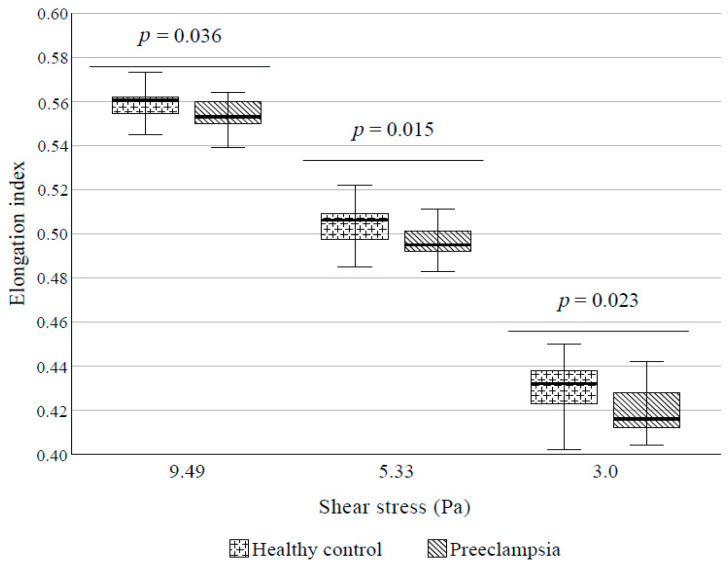
Deformability (EI values) at medium shear stresses in preeclampsia and control group. Box plot diagrams show the median with interquartile ranges and the minimum and maximum values.

We found statistically significant deterioration of erythrocyte deformability under medium shear stresses (9.49, 5.33, and 3 Pa) at PE diagnosis compared to controls (Figure 4).

Analyzing changes within groups, we found that the RBC deformability improved until postpartum 72 h in PE compared to the values at diagnosis or during delivery (Supplementary Table S1a). Our results did not show any elevation in EI values in controls. Considerably decreased EI values were found at high shear stress during delivery, but after 72 h, the deformability returned to similar values as measured at enrolment (Supplementary Table S1b).

### 4.5. Indicators of RBC Aggregation for Preeclampsia Diagnosis

**Figure 5:**
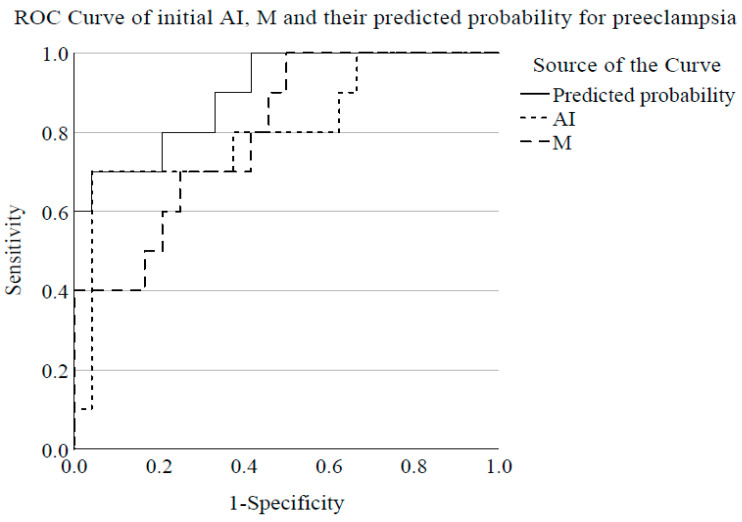
ROC curve for preeclampsia comparing initial AI and M values per se measured at diagnosis or enrolment and their combination expressed as a predicted probability.

ROC analysis was carried out with maternal aggregation parameters in the first investigated time point to test the diagnostic power for PE. The analysis of initial AI indicated a cutoff point of 69.4% for PE with an AUC of 0.837 [0.684–0.990] (*p* = 0.001) (sensitivity = 83.3%; specificity = 62.5%). ROC analysis of initial M values showed an AUC of 0.750 [0.576–0.924] (*p* = 0.019) and indicated a cutoff of 8.39 (sensitivity = 90.9%; specificity = 50%). The predicted probabilities from the combination of initial AI and M variables produced by binary logistic regression analysis showed slightly increased AUC with 0.900 ([0.789–1.000] *p* < 0.001) comparing the ones of AI or M value per se as classifiers for PE (Figure 5).

## 5. Discussion

Our research findings are intended to emphasize and confirm the role of maternal hemorheological alterations in pathophysiological processes affecting microcirculation in early-onset PE. Our investigations focused mainly on peripartum alterations of RBC properties including their aggregation and deformability. The complex pathophysiological background of early-onset PE including inflammatory, genetic, immunological, hemodynamic changes, oxidative stress, and disturbed trophoblastic invasion finally leads to vascular endothelial damage, vasospasm, and hypertension. These pathophysiological mechanisms alter the biophysical properties of RBCs including their aggregation and deformation abilities. Deterioration of these factors (i.e., elevated RBC aggregation and reduced deformability) leads to increased viscosity, which may hinder the proper tissue perfusion in the intervillous space of the placenta, and hence the erythrocytes are not able to ensure adequate oxygen delivery to the fetus. The damaged erythrocytes may enhance the progression of the endothelial dysfunction and maternal circulatory disorders.

### 5.1. Routinely Measured RBC Laboratory Parameters

During normal pregnancy, total blood volume, plasma volume, and RBC mass are extensively increasing. Additionally, the plasma volume is elevating proportionally more than the RBC mass, resulting in lower hemoglobin concentrations from physiological hemodilution [16]. However, we observed slightly elevated RBC count and hemoglobin values in early-onset PE, which are in line with previous findings [17]. Initial MCV was slightly decreased in our PE group compared to that in controls, contrary to previous investigations, according to whom significantly increased MCV occurred in PE [17,18]. Importantly, in these studies, the mean gestational age was higher in the PE and control groups as well than in our study. According to a recent publication analyzing the osmotic and mechanical stability of erythrocytes, it is suggested that erythrocytes with lower volume and lower hemoglobin content are osmotically more stable. They assumed that lower MCV values could be a mechanism of compensatory mechanical selection, which is beneficial in PE [19]. However, the desired range of MCV throughout pregnancies complicated by PE remains unclear.

### 5.2. RBC Aggregation

According to our results, elevated initial maternal AI and M values proved to be the most promising indicators of PE with high sensitivity and acceptable specificity. Moreover, we gained more favorable AUC by their combination compared to that in their individual analysis. The elevated RBC aggregation at PE diagnosis compared to that in controls refers to impaired microcirculation in early-onset PE, which is also supported by the positive relationship between RBC aggregation at PE diagnosis and gestational age at delivery. This association suggests that the longer the pathologic pregnancy persists, the worse the AI values are, reflecting enhanced maternal RBC aggregation. We did not observe a significant relationship in healthy pregnancies concerning their AI value and their gestational age at enrolment, contrary to PE. These observations suggest that in normal pregnancy, the gestational age alone did not influence AI values, this association was specific to PE. The results of multiple regression analysis also supported the significant relationship of the AI values and the gestational weeks at delivery. It should be noted that initial AI values in both groups were independent of hemoglobin values, so they did not affect our measurement results in either group. Analyzing the changes within the groups, we can conclude that RBC aggregation increased within the first 72 postpartum hours in healthy pregnancies. Contrary, continuously elevated RBC aggregation was observed in PE, and we did not find a significant difference between the three investigated time points. 

The cesarean section rate was significantly lower in the healthy control group, as expected. The hypothesis was raised that the difference in the mode of delivery may influence the results measured at delivery and in the postpartum period, since different physiological processes occur according to the mode of delivery regardless of the underlying obstetric disease. In contrast, our subgroup analyses showed that RBC aggregation values were not significantly affected by this variable. After excluding mothers who gave birth in a natural way, similar differences were revealed between the control and preeclamptic groups.

The RBC aggregation activity depends on the plasma protein levels and intrinsic properties of the erythrocytes such as alterations affecting the cell membrane [20]. In PE, changes of cellular factors are suggested to be more responsible for the pronounced aggregation propensity [10]. Increased RBC aggregation can be attributed to the reduced sialic acid content of the cell membrane that weakens repulsive forces [21] and conformational changes of the membrane enhancing erythrocyte aggregation [22]. A most recent study also confirmed decreased erythrocytic membrane anionic charge due to the reduced sialic acid content of the erythrocyte cell membrane in women with PE/eclampsia, which may be responsible for the aggregation of erythrocytes in these conditions [23].

Although we did not measure blood coagulation substances in the present study, the increased RBC aggregation in healthy mothers in the postpartum period compared to values before delivery is thought to be mainly due to increasing plasma protein (e.g., fibrinogen, immunoglobulin M, and alpha 2-macroglobulin) levels, since these are known to enhance the aggregating activity of RBCs [24]. During normal pregnancy, the RBC aggregation increases despite hemodilution, and this observation has been previously mainly explained via the nearly linear increase of fibrinogen synthesis in the liver [24]. In the third trimester of normal pregnancy, the physiological reserve of blood coagulation substances is accumulating to maintain a hypercoagulability state, probably enhancing erythrocyte aggregation as well, such changes are more pronounced after childbirth to prevent postpartum hemorrhage. With the progression of PE, the locally enforced coagulation state leads to the consumption of the coagulation substances, resulting in an overall low coagulation state. Compared with healthy pregnant women, PE patients had decreased fibrinogen values and a relatively hypocoagulable state was described [25]. Although literature data show elevated fibrinogen levels in healthy pregnancies, in our PE group, the RBC aggregation was still higher throughout the study period since aggregation is influenced not only by the external (plasmatic components such as fibrinogen) but also by the intrinsic properties of RBCs, which are likely to play a greater role in PE [10]. It is also conceivable that the longer time interval between enrolment and delivery in the control group (Table 1) made the physiological increase of the RBC aggregation values possible.

### 5.3. RBC Deformability

Initial EI values at medium shear stresses were decreased in PE reflecting altered RBC deformability. The reason for reduced RBC deformability in PE is suggested to be chronic inflammation and hypoxia, which leads to an increased concentration of free radicals inducing changes in RBC membrane properties and subsequent increase of intracellular Ca^2+^ [26]. In addition, an increased Ca^2+^ pump activity leads to adenosine triphosphate (ATP) depletion in RBCs resulting in poor deformability [27].

The relatively rapid improvement of deformability in the postpartum period in PE may presumably occur due to the termination of gravidity, which has maintained the disease. It can, therefore, be hypothesized that RBC deformability may be a sensitive and early marker of the normalization of maternal microcirculation after delivery. The deformation ability of erythrocytes depends on complex biophysical properties: the internal cell viscosity, membrane viscoelasticity (e.g., spectrin–F-actin network; phospholipid bilayer; pump activity, ATP pool, intracellular Ca^2+^ concentration), surface–volume ratio, and erythrocyte morphology. The consequence of increased internal cell viscosity or membrane rigidity or elevated surface–volume ratio is reduced elongation ability [28]. External factors may provoke rapid change in a number of these characteristics leading to alteration in the deformability. In the study of Schauf et al., reduced RBC deformability was described in pregnancies complicated by PE and/or IUGR. They reported that deformability values returned within 5 days after delivery to the RBC deformability observed in early pregnancy, and these mentioned changes were already visible on the first and second postpartum days [29]. These results are in line with our findings. They did not discuss the reason for deformability improvement in the postpartum period; however, they suggested that intracellular Ca^2+^ concentration plays a key role in the regulation of RBC mechanical properties. An increase in intracellular Ca^2+^ concentration leads to a reduction of RBC deformability. It was shown that intracellular Ca^2+^ concentration is elevated in PE, which might explain the reduction of RBC deformability [30]. It was previously described that the diminution in the activity of the RBC Ca^2+^-ATPase enzyme in PE is related to an enhanced level of lipid peroxidation of the plasma membranes [31]. The placenta is thought to be the central organ that regulates this condition and the major source of reactive oxygen species during pregnancy, which deteriorate the erythrocyte membrane’s properties [32]. At the same time, when delivery occurs, the maintaining cause of the disease is also removed. Consequently, it can be suggested that a relatively rapid change may happen in erythrocyte membrane or cytoskeletal properties (e.g., due to the improved pump activity or phospholipid bilayer status). However, these are only assumptions, and no precise explanations have been found in the literature to date.

### 5.4. Previous Data Concerning RBC Properties

Recent studies on RBC deformability and aggregation in PE seem to be in conflict. Some authors found decreased erythrocyte deformability in PE [12,17,33] and uteroplacental hypoperfusion with subsequent RBC membrane damage has also been described [27]. Others did not observe any significant deformability alteration in PE [34,35]. Tranquilli et al. reported increased erythrocyte aggregation in PE [22], while other authors did not observe significant deterioration [36,37]. In line with our observations, Heilmann et al. found enhanced RBC aggregation and reduced deformability by high shear stress in patients with severe PE, suggesting that hemorheological parameters play an important role in the microcirculation of the intervillous space of the placenta [17]. 

Most of the studies dealing with hemorheological alterations in PE were published more than 2–3 decades ago. As in the case of PE definitions, the measurement methods and interpretation of results may have changed since then; therefore, it is necessary to re-evaluate these results. Moreover, the diagnostic criteria for the early-onset type have been broadened and clarified [3]. The distinction between the early- and late-onset forms is increasingly recognized since they have a different pathophysiological background and clinical features, thus they should be investigated completely separately. Early-onset PE is suggested to mainly originate from defective placentation, whilst the late-onset form may develop due to maternal genetic predisposition to cardiovascular and metabolic disease [38]. Therefore, it would be desirable to treat the two types separately from a hemorheological point of view as well.

Regarding RBC aggregation, previous investigations mostly applied a Myrenne aggregometer per se [12,17], while only a few reported results were measured by LORCA [36]. To the best of our knowledge, there are no data in the literature that examine these two measurement methods simultaneously in early-onset PE as we have done. It can be seen from our report that the two types of measurement methods gave similar results in terms of RBC aggregation, moreover, their combination would even raise the diagnostic power.

Although in this study we intended to evaluate exclusively the erythrocyte properties, it is also desirable to mention the role of the platelets in PE, as they are the other most important cellular elements of blood. In addition, platelet aggregation activity is a relevant hemorheological parameter as well. However, we would also like to note that platelet characteristics do not influence the RBC aggregation and deformability measurement results. A recent systematic review suggests higher platelet activation and lower platelet aggregation or no difference [39]. However, results concerning platelet aggregation in PE are controversial. Platelet activation, aggregation, and adhesion markers are also essential components of the pathogenesis of PE, which is also supported by the efficacy of aspirin prophylaxis. However, further investigations are required to prove the predictive value in PE due to the conflicting results.

### 5.5. Screening

In the past decade, extensive efforts have been made to develop an efficient screening algorithm to identify high-risk patients [40]. Currently, the best screening model in the first trimester combines multiple examinations containing maternal risk factors, comorbid conditions, physical parameters, mean arterial pressure, placental growth factor level, and uterine artery pulsatility index. Women identified to be at high risk should receive 150 mg acetylsalicylic acid prophylaxis daily commencing at 11–14^+6^ weeks of gestation until either 36 weeks, when delivery occurs, or when the disease is diagnosed [41].

Determination of factors and specific biomarkers used in the current screening algorithm is difficult to perform, time-consuming, and contains costly measurements requiring the involvement of qualified professionals. In contrast, we investigated a cheap method with easy implementation and indicators that are quantitative, objective, and can be blinded to other clinical characteristics. Excluding the purchase of the instruments, hemorheological test costs are minimal and can be easily performed without special training. The above-detailed measurements require a quite small amount of blood as a sample and provide quickly obtainable results. Therefore, further investigations are suggested to reveal whether RBC aggregation and deformability parameters, especially AI and M values really possess the potential for susceptibility or risk biomarkers in the early stage of PE before the onset of symptoms. Moreover, their inclusion in the screening algorithm should be considered. 

### 5.6. Strengths and Limitations

The strength of our study is the prospective, case-control design with repeated blood sampling in the peripartum period to evaluate the kinetics and changes of RBC properties. Using two methods together (Myrenne, measuring the increase of light transmission through plasma gaps between RBC aggregates, and LORCA, detecting laser backscattering from the RBC aggregates) provides more reliable information on RBC aggregation properties. The low total number of enrolled patients and its single-center nature with local treatment strategies and guidelines could limit the generalizability of our findings.

## 6. Conclusions

The most remarkable findings of our research are the potential diagnostic power of elevated AI and M index reflecting enhanced RBC aggregation in early-onset PE. A positive linear relationship was observed between initial AI and gestational age at birth in PE. Reduced initial EI values were found in PE, reflecting impaired erythrocyte deformability at diagnosis, the values of which were improved within three days after delivery. RBC properties could help in the prognostication of early-onset PE, but further investigations are warranted to confirm the prognostic role before the onset of symptoms.

## Figures and Tables

**Table 1:** Maternal and neonatal demographic and clinical data.

Maternal	**Baseline Characteristics**	**Preeclampsia**	**Control**	***p*-Value**
	Age (years)	29.08 ± 2.13	30.42 ± 1.39	0.589
	Gestation age at first sampling (weeks)	29.69 ± 0.67	28.71 ± 0.48	0.240
	Systolic blood pressure at admission (mmHg)	160 (146–175)	120 (119–130)	<0.001
	Diastolic blood pressure at admission (mmHg)	100 (90–110)	80 (70–80)	<0.001
	Heart rate at admission (/min)	82 (80–95)	82 (77–88)	0.320
	Body height (m)	1.63 ± 0.06	1.67 ± 0.07	0.094
	Body weight (kg)	78 (70–87)	80 (70–86)	0.824
	BMI (kg/m^2^)	30.1 (26.8–33.5)	28.2 (25.7–30.2)	0.227
	Change in body weight (kg)	10 ± 7	13 ± 4	0.132
	Mode of delivery: cesarean section (n; %)	13; 100%	9; 37.5%	<0.001
	Length of hospital stay (day)	8 (6–15)	4 (4–5)	<0.001
Neonatal	Gestational age at birth (weeks)	30.23 ± 0.86	39.06 ± 0.28	<0.001
	Birth weight (gram)	1355.83 ± 157.69	3420.00 ± 89.04	<0.001
	Birth length (cm)	36.67 ± 1.64	49.76 ± 0.80	<0.001
	Head circumference (cm)	27.81 ± 1.01	34.24 ± 0.35	<0.001
	Shoulder width (cm)	28.09 ± 1.30	36.88 ± 0.57 cm	<0.001
	Apgar 1	7.0 (6.0–8.0)	9.0 (9.0–9.0)	<0.001
	Apgar 5	9.0 (8.0–9.0)	10.0 (10.0–10.0)	<0.001
	IUGR (n; %)	7; 53.8%	0	<0.001

The results were expressed as the mean value ± standard deviation of the mean or median and interquartile range. BMI: body mass index; IUGR: intrauterine growth restriction.

## Data Availability

All data relevant to the study are included in the article or uploaded as Supplementary Materials.

## Supplementary Materials

The following are available online at https://www.mdpi.com/article/10.3390/cells10102714/s1. Supplementary material for 1. Introduction section; Supplementary material for 3. Materials and methods section: Supplementary Figure S1. and Supplementary Figure S2; Supplementary material for 4. Results section: Supplementary Figure S3. and Supplementary Figure S4. and Supplementary Figure S5. and Supplementary Table S1a. Elongation index on different shear stresses in PE group; Supplementary Table S1b. Elongation index on different shear stresses in healthy pregnant women.
