# Supplementary material for: Peripartum Investigation of Red Blood Cell Properties in Women Diagnosed with Early-Onset Preeclampsia
Source: Cells. 2021 Oct 10;10(10):2714. doi: 10.3390/cells10102714 (PMC8534376; doi:10.3390/cells10102714)
Supplement: Supplementary file 1 [file cells-10-02714-s001.zip › cells-1363614-supplementary.pdf]

Supplementary material

Peripartum investigation of red blood cell properties in women diagnosed with early-onset preeclampsia

Beata Csiszar[1,2], Gergely Galos[3], Simone Funke[4], Dóra Kinga Kevey[4], Matyas Meggyes[5,2], Laszlo Szereday[5,2], Peter Kenyeres[6,2], Kalman Toth[6,2], Barbara Sandor[6,2]

[1]University of Pécs, Medical School, Department of Anaesthesiology and Intensive Therapy, Pécs, Hungary
[2]Szentágothai Research Centre, Pécs, Hungary
[3]University of Pécs, Medical School, Pécs, Hungary
[4]University of Pécs, Medical School, Department of Obstetrics and Gynaecology, Pécs, Hungary
[5]University of Pécs, Medical School, Department of Medical Microbiology and Immunology, Pécs, Hungary
[6]University of Pécs, Medical School, 1st Department of Medicine

Corresponding author:
Beata Csiszar, MD
University of Pécs, Medical School, 1st Department of Medicine
7624 Pecs, 13 Szigeti Street, Hungary
E-mail: csiszar.beata@pte.hu

Supplementary material for 1. Introduction section:

The mechanism of erythrocyte aggregation and deformability

Erythrocyte aggregation means reversible rouleaux formation of red blood cells (RBC) mostly due to bridging action of large molecular weight proteins (fibrinogen and immunoglobulins) in plasma. There are two theories concerning the background of the RBC aggregation process. According to the "*bridging theory*" RBC aggregation is caused by macromolecules enhancing the connection of the cells, while the "*depletion layer theory*" suggests that the diminished macromolecular concentration creates an osmotic gradient between two RBCs leading to cell-cell interactions [1]. Under low shear conditions, erythrocytes first arrange into linear and then three-dimensional structures similar to a stack of coins, but with increasing shear forces the process becomes reversible leading to disaggregation of erythrocytes. The formation also occurs in vitro in a plasma-free isoosmotic environment, thus not only the components of plasma but also intracellular factors are involved in the process. Previous studies have shown that aggregation is influenced by extracellular factors such as the concentration of fibrinogen and other polymers (e.g. dextran) in the plasma and by adhesion molecules on the cell surface

[1]. RBC aggregation is a determinant of blood viscosity, and its increase enhances friction between fluid plates, thus adversely affecting both macro-, and microcirculation.

Erythrocyte deformability is described as the ability of RBC to adapt to mechanical forces and so be able to cross over narrow capillaries and ensure sufficient tissue oxygenation. Normal RBCs are biconcave in shape, 7-8 μm in diameter and around 2 μm thick. In contrast, the lumen of the capillaries in human tissues can be 3-5μm in diameter. The passage of erythrocytes through such capillaries requires the above-mentioned major modification of their structure, the so-called RBC deformability i.e. their ability to change shape. Deformability is determined by the internal cell viscosity, RBC membrane properties, morphology, and surface-volume ratio. However, other extrinsic factors in the circulation also affect this property, such as serum protein concentration. Rigid RBCs are unable to deform in response to shear forces, consequently, the deformation capacity is reduced, thus higher viscosity values can be measured, especially when higher shear stresses are applied [2]. Deformability also affects the macro- and microcirculation, but in inverse proportion. Its elevation allows RBCs in high-volume vessels to assume a streamlined shape, thereby reducing viscosity. At the capillary level, this property helps to pass through blood vessels of a much narrower lumen than their diameter.

To summarise, elevated RBC aggregation and decreased deformability can adversely affect the hemorheological properties and impair tissue perfusion.

Supplementary material for 3. Materials and methods section:
Measurement techniques and instruments for evaluating erythrocyte aggregation and deformability

RBC aggregation was determined with two instruments employing different approaches of the method of *syllectometry* which means measuring light intensity changes due to erythrocyte aggregation. Myrenne aggregometer using infrared light transmission, while LORCA aggregometer operating with laser backscattering.

Myrenne aggregometer (model MA-1, Myrenne GmbH, Roetgen, Germany; Supplementary Fig. 1.) applies and measures the infrared light transmission through an erythrocyte suspension between a transparent plate and a cone. This technique is based upon the increase of light transmission through plasma gaps between the rouleaux aggregates or rouleaux-rouleaux complexes made up of RBCs. The cone plate system rotates the injected 30 μl blood sample for 10 seconds at high shear stress (at 600 s^{-1}), to disperse all pre-existing cell aggregates. Then the system stops instantly and measures in stasis (M mode), or the shear is reduced to 3 s^{-1} and measures at lower shear stress (M1 mode) to stimulate aggregation, what leads to increasing light transmission proportional to the rate of RBC aggregate formation during stasis (M index) or at low shear (M1 index). The aggregation is determined by the quantity of infrared light transmission measured by photosensors at room temperature. The two dimensionless indices (M, M1) increase with enhanced erythrocyte aggregation [3].

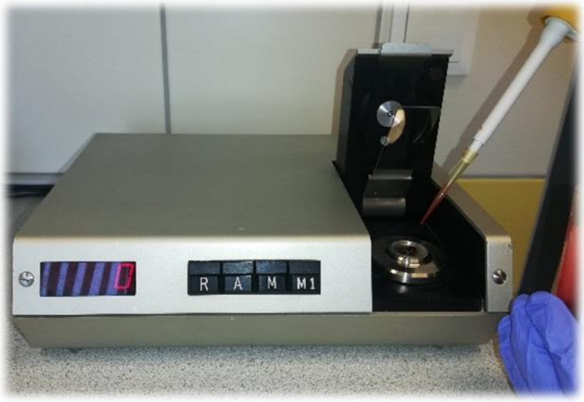

Supplementary Figure S1. *Myrenne aggregometer*

The **Laser-assisted Optical Rotational Cell Analyzer** (LORCA - R&R Mechatronics, Hoorn, Netherlands; Supplementary Fig. 2.) determines the erythrocyte aggregation by detecting laser backscattering from the RBC aggregates. 1 ml blood sample is injected between the outer, rotating cylinder, and the inner, static cylinder of the instrument and RBCs are disaggregated at a high shear rate (500 s^{-1}) at 37 °C. Then the motor rapidly stops. The intensity of reflected light is measured for 120 seconds and is plotted as a function of time. The aggregation index (AI) and the aggregation half-time ($t_{½}$ - which is the time required to reach half of the maximum aggregation) are calculated from the syllectogram during the first 10 seconds of the measurement. Further parameter describing red blood cell aggregation is the threshold shear rate (γ) which is defined as the smallest shear rate required for the complete disaggregation of RBCs [4].

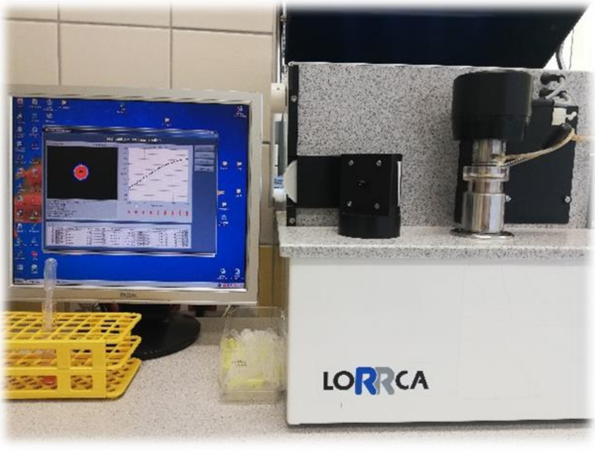

Supplementary Figure S2. *LORCA aggregometer*

In this study erythrocyte deformability on different shear stresses was determined by LORCA as well. 20 µl blood sample was diluted in a viscous medium called polyvinilpyrrolidon and injected between the cylinders. A laser-diode is projected through the fluid, the light diffracts on the RBCs resulting in a diffraction pattern on a diaphragm. This will be analysed by a video camera and a computer system. As a result of the applied increasing shear stress RBCs will be elongated and the diffraction pattern is

changing from circular to elliptical shape. Based on the measurements we could express RBC deformability as elongation index (EI) given at each shear stress [5].

Supplementary material for 4. Results section:

The impact of the mode of delivery

All patients diagnosed with preeclampsia (n=13; 100%) and 9 women (37.5%) in the control group had Caesarean section. The hypothesis that the difference in the mode of delivery may influence the results measured at delivery and in the postpartum period seems logical, since different pathophysiological processes occur according to the mode of delivery. Therefore, we performed additional statistical analysis to exclude the potentially disturbing impact of the mode of delivery. In these figures, we analysed the maternal erythrocyte aggregation index (AI) and M index values between Ceasarean section (n=9) and normal delivery mode (n=15) within the group of healthy pregnant women. We observed no significant difference concerning AI or M values.

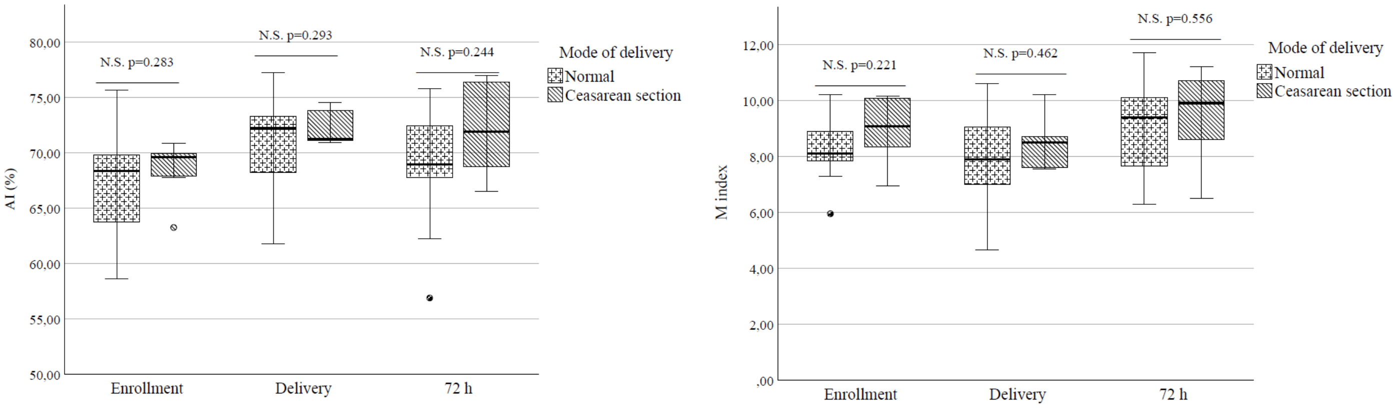

Supplementary Figure S3. *AI and M index according to the mode of delivery in healthy pregnancies*

Moreover, we evaluated these parameters comparing the results of the preeclampsia group (n=13) with the subgroup of healthy pregnant women with Caesarean section (n=9).

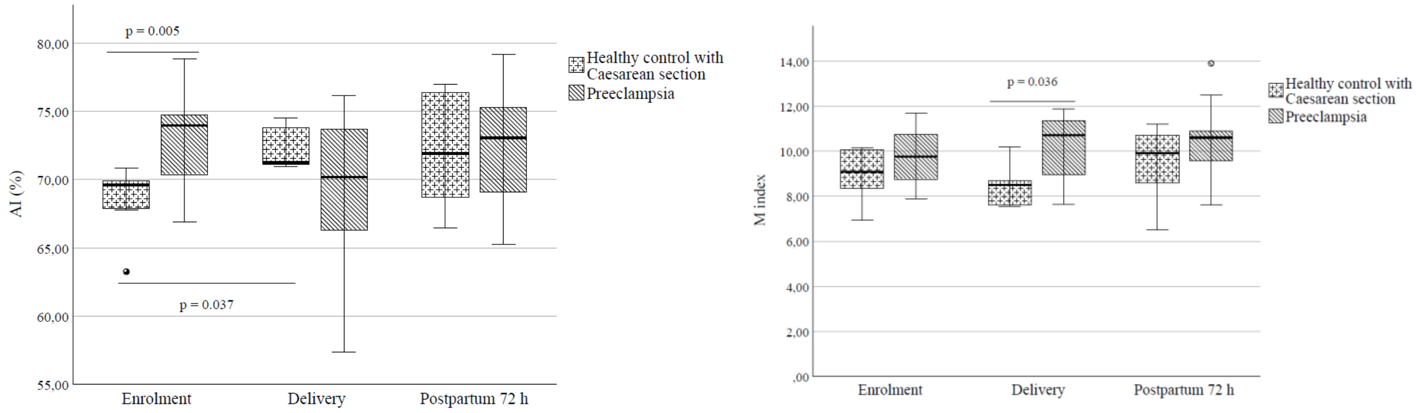

Supplementary Figure S4. *AI and M index in preeclampsia and healthy pregnancies with Caesarean section subgroup*

The above figures show that comparing the subgroup of the healthy women with Caesarean section with women suffering from preeclampsia, we obtained similar results as before. These findings confirm that our results were not disturbed by the mode of delivery.

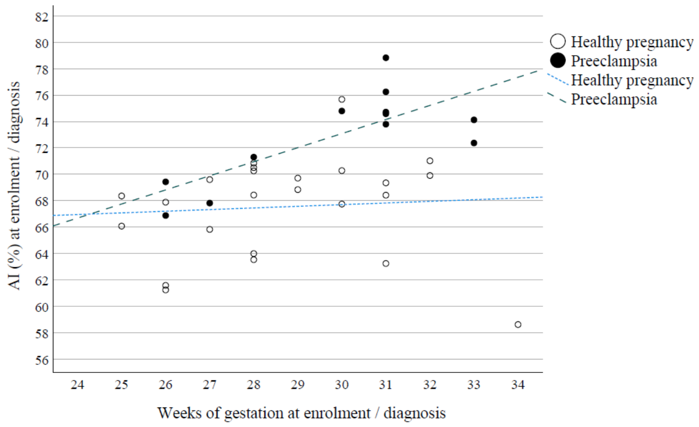

Supplementary Figure S5. *AI measured at enrolment and weeks of gestation at enrolment in healthy and preeclampsia groups*

Supplementary Table S1.a. *Elongation index on different shear stresses in PE group*

	Time of blood sampling			p-values		
Shear stress	Diagnosis (1)	Delivery (2)	Postpartum 72 h (3)	1 vs. 2	2 vs. 3	1 vs. 3
EI, 30.00 Pa	0.625 ± 0.006	0.626 ± 0.006	0.626 ± 0.005	0.724	0.469	0.644
EI, 16.87 Pa	0.599 ± 0.007	0.600 ± 0.006	0.602 ± 0.006	0.606	0.109	0.189
EI, 9.49 Pa	0.554 ± 0.008	0.555 ± 0.008	0.600 ± 0.010	0.769	**0.015***	**0.048***
EI, 5.33 Pa	0.496 ± 0.009	0.499 ± 0.010	0.505 ± 0.012	1.000	**0.008***	**0.018***
EI, 3.00 Pa	0.421 ± 0.011	0.423 ± 0.012	0.431 ± 0.015	0.916	**0.007***	**0.015***
EI, 1.69 Pa	0.329 ± 0.017	0.332 ± 0.016	0.341 ± 0.019	0.901	**0.021***	**0.009***
EI, 0.95 Pa	0.226 ± 0.019	0.229 ± 0.020	0.239 ± 0.023	0.720	**0.016***	**0.018***
EI, 0.53 Pa	0.122 ± 0.023	0.126 ± 0.025	0.134 ± 0.028	0.530	**0.046***	**0.026***
EI, 0.30 Pa	0.045 ± 0.028	0.047 ± 0.018	0.053 ± 0.029	0.690	0.314	0.321

The results were expressed as the mean value ± standard deviation of the mean. *p<0.05

Supplementary Table S1.b. *Elongation index on different shear stresses in healthy pregnant women*

	Time of blood sampling			p-values		
Shear stress	Enrolment (1)	Delivery (2)	Postpartum 72 h (3)	1 vs. 2	2 vs. 3	1 vs. 3
EI, 30.00 Pa	0.622 ± 0.008	0.620 ± 0.008	0.624 ± 0.007	**0.037***	**0.010***	0.231
EI, 16.87 Pa	0.600 ± 0.006	0.598 ± 0.006	0.602 ± 0.008	**0.018***	**0.032***	0.489
EI, 9.49 Pa	0.559 ± 0.007	0.557 ± 0.006	0.561 ± 0.010	**0.022***	0.076	0.693
EI, 5.33 Pa	0.504 ± 0.009	0.504 ± 0.008	0.507 ± 0.012	0.240	0.092	0.450
EI, 3.00 Pa	0.430 ± 0.011	0.430 ± 0.012	0.433 ± 0.018	0.195	0.143	0.716
EI, 1.69 Pa	0.339 ± 0.016	0.340 ± 0.017	0.344 ± 0.025	0.291	0.063	0.600
EI, 0.95 Pa	0.235 ± 0.021	0.240 ± 0.022	0.242 ± 0.030	0.891	0.373	0.516
EI, 0.53 Pa	0.130 ± 0.023	0.135 ± 0.024	0.137 ± 0.031	0.862	0.668	0.442
EI, 0.30 Pa	0.050 ± 0.029	0.055 ± 0.018	0.048 ± 0.029	0.833	0.389	0.694

The results were expressed as the mean value ± standard deviation of the mean. *p<0.05

References

1. Chien, S.; Sung, L. A., Physicochemical basis and clinical implications of red cell aggregation. *Clinical Hemorheology and Microcirculation* **1987,** *7*, 71-91.
2. Mohandas, N.; Clark, M. R.; Jacobs, M. S.; Shohet, S. B., Analysis of factors regulating erythrocyte deformability. *J Clin Invest* **1980,** *66* (3), 563-73.
3. Vayá, A.; Falcó, C.; Fernández, P.; Contreras, T.; Valls, M.; Aznar, J., Erythrocyte aggregation determined with the Myrenne aggregometer at two modes (M0, M1) and at two times (5 and 10 sec). *Clin Hemorheol Microcirc* **2003,** *29* (2), 119-27.
4. Hardeman, M. R.; Dobbe, J. G.; Ince, C., The Laser-assisted Optical Rotational Cell Analyzer (LORCA) as red blood cell aggregometer. *Clin Hemorheol Microcirc* **2001,** *25* (1), 1-11.
5. Rabai, M.; Detterich, J. A.; Wenby, R. B.; Hernandez, T. M.; Toth, K.; Meiselman, H. J.; Wood, J. C., Deformability analysis of sickle blood using ektacytometry. *Biorheology* **2014,** *51* (2-3), 159-70.
